# Nanoemulsified Essential Oil of *Melaleuca leucadendron* Leaves for Topical Application: In Vitro Photoprotective, Antioxidant and Anti-Melanoma Activities

**DOI:** 10.3390/ph17060721

**Published:** 2024-06-02

**Authors:** Lucas Resende Dutra Sousa, Maria Luiza da Costa Santos, Larissa Silva Sampaio, Clarisse Gaëlle Faustino, Mérine Lauriane Loïce Guigueno, Kátia Michelle Freitas, Miriam Teresa Paz Lopes, Gabriela Cristina Ferreira Mota, Viviane Martins Rebello dos Santos, Janaína Brandão Seibert, Tatiane Roquete Amparo, Paula Melo de Abreu Vieira, Orlando David Henrique dos Santos, Gustavo Henrique Bianco de Souza

**Affiliations:** 1 Laboratório de Fitotecnologia, Escola de Farmácia, Universidade Federal de Ouro Preto, Campus Morro do Cruzeiro, Ouro Preto 35400-000, MG, Brazil; lucasresendedutrasousa@gmail.com (L.R.D.S.); maria.lcs@aluno.ufop.edu.br (M.L.d.C.S.); larissa.sampaio1@aluno.ufop.edu.br (L.S.S.); 2 Programa de Pós-Graduação em Ciências Farmacêuticas, CiPharma, Universidade Federal de Ouro Preto, Ouro Preto 354000-000, MG, Brazil; tatianeroquete@yahoo.com.br; 3 École de Biologie Industrielle, 49 Avenue des Genottes, 95800 Cergy, France; c.faustino@hubebi.com (C.G.F.); m.guigueno@hubebi.com (M.L.L.G.); 4 Departamentos de Farmacologia, Instituto de Ciências Biológicas, Universidade Federal de Minas Gerais, Av Antônio Carlos 6627, Belo Horizonte 31270-901, MG, Brazil; artemisk_farma@yahoo.com.br (K.M.F.); mtpl.ufmg@gmail.com (M.T.P.L.); 5 Laboratório de Produtos Naturais e de Síntese Orgânica, Instituto de Ciências Exatas e Biológicas, Universidade Federal de Ouro Preto, Campus Morro do Cruzeiro, Ouro Preto 35400-000, MG, Brazil; gabriela.mota@aluno.ufop.edu.br (G.C.F.M.); vivianesantos@ufop.edu.br (V.M.R.d.S.); 6 Laboratório de Patologia e Controle Microbiano, Universidade de São Paulo (USP-ESALQ), Piracicaba 13418-900, SP, Brazil; janainaseibert@usp.br; 7 Laboratório de Química Medicinal e Bioensaios, Escola de Farmácia, Universidade Federal de Ouro Preto, Campus Morro do Cruzeiro, Ouro Preto 35400-000, MG, Brazil; 8 Laboratório de Morfopatologia, Núcleo de Pesquisas em Ciências Biológicas, Universidade Federal de Ouro Preto, Campus Morro do Cruzeiro, Ouro Preto 35400-000, MG, Brazil; paula@ufop.edu.br

**Keywords:** antioxidant, essential oil, *Melaleuca leucadendron*, melanoma, nanoemulsion, rheological behavior, sun protect factor, transmission electronic microscopy

## Abstract

Melanoma, primarily caused by solar ultraviolet (UV) radiation, can be prevented by the use of sunscreens. However, the use of synthetic sunscreens raises environmental concerns. Natural compounds with antioxidant photoprotective properties and cytotoxic effects against cancer cells can be promising for the prevention and treatment of melanoma with less environmental effect. This study focuses on *Melaleuca leucadendron* essential oil (EO) for photoprotection and antitumor applications. EO was hydrodistilled from *M. leucadendron* leaves with a 0.59% yield. Gas chromatography–mass spectrometry detected monoterpenes and sesquiterpenes. Nanoemulsions were prepared with (NE-EO) and without EO (NE-B) using the phase inversion method, showing good stability, spherical or oval morphology, and a pseudoplastic profile. Photoprotective activity assessed spectrophotometrically showed that the NE-EO was more effective than NE-B and free EO. Antioxidant activity evaluated by DPPH and ABTS methods indicated that pure and nanoemulsified EO mainly inhibited the ABTS radical, showing IC_50_ 40.72 and 5.30 µg/mL, respectively. Cytotoxicity tests on L-929 mouse fibroblasts, NGM human melanocyte, B16-F10 melanoma, and MeWo human melanoma revealed that EO and NE-EO were more cytotoxic to melanoma cells than to non-tumor cells. The stable NE-EO demonstrates potential for melanoma prevention and treatment. Further research is required to gain a better understanding of these activities.

## 1. Introduction

Melanoma is a serious type of skin cancer that develops from melanocytes located in the basal layer of the epidermis, and it has a significant global impact [1]. In countries with a predominantly fair-skinned population, the incidence of melanoma is increasing. In 2020, the estimated global incidence of the disease was 325,000 cases, representing a 41% increase from 230,000 cases in 2012 [2,3]. It is projected that if the current rate of increasing cases remains stable, the global burden of melanoma will increase to 510,000 new cases and 96,000 deaths by 2040 [4]. In this context, early diagnosis and adequate treatment are of paramount importance in order to enhance survival rates [5,6,7].

The current standard of care for melanoma is surgical removal, particularly in the early stages of the disease [8]. Other treatment options include immunotherapy and targeting cells with mutations in the BRAF gene. However, these therapeutic options are costly and often inaccessible to patients [8,9]. Surgery can result in the formation of unwanted scars, while medications are associated with a range of side effects, including dermatological problems and serious complications such as heart problems and allergic reactions [9,10].

Exposure to ultraviolet (UV) light from the sun is identified as the main risk factor linked to the development of melanoma, although the interaction between this factor and genetics, melanin, and the wavelengths of UV rays is a crucial aspect. UV radiation can damage deoxyribonucleic acid (DNA), triggering the formation of photoproducts. UVB radiation with a wavelength of 280–320 nm is considered highly genotoxic, whereas UVA with a wavelength of 320–400 nm is more prevalent in the environment and less photon genotoxic [11,12].

Regular use of sunscreen (with a sun protection factor (SPF) of 30 or higher) has been demonstrated to have a positive impact on the reduction of long-term melanoma rates, emphasizing its significance in protecting the skin against the damage caused by sun exposure [1]. However, concerns have been raised regarding synthetic sunscreens, such as oxybenzone and octinoxate, which can be assimilated through the skin and disturb the human endocrine system, disrupting hormonal balance. Furthermore, the adverse impact of these filters on marine ecosystems and coral life is well-documented, and efforts to explore alternative, environmentally friendly sunscreens—particularly those derived from natural sources—are strongly encouraged [13].

Currently, there is increasing interest in integrating natural ingredients into photoprotective formulations due to their established antioxidant effects and capacity to absorb UV radiation [14,15]. These natural substances have demonstrated the potential to enhance the efficacy of sunscreens [16]. In this context, essential oils emerge as promising options, as they have already demonstrated the ability to enhance the SPF of formulations containing synthetic filters, thereby allowing for a reduction in their content in new formulations [17]. In addition to the increasing benefits of protection against sun damage by improving the SPF, it was reported that certain natural ingredients have demonstrated antioxidant activity and cytotoxic properties against skin cancer cells [18]. This could potentially play a significant role in preventing the development of cutaneous melanoma.

*Melaleuca leucadendron* (Myrtaceae), along with other species within the same genus, has acquired recognition for the antioxidant and cytotoxic potential of the essential oil from their leaves. Terpenes such as 1,8-cineole, α-terpineol, α-pinene, limonene, globulol, and guaiol were identified as potent antioxidants in the essential oil of this species [18], while α-terpineol, terpinolene, terpinen-4-ol, hinesol, and α-pinene compounds were found to possess previously reported antitumor activity [19,20,21]. These data show that this oil can be interesting for research in the prevention and treatment of melanoma.

Nanoemulsions, as nanostructured systems, offer valuable alternatives for the incorporation of essential oils in pharmaceutical formulations [22,23]. These systems possess stability, biodegradability, and biocompatibility, exemplifying efficacy through varied administration routes, inclusive of dermal administration, thereby reducing side effects and prolonging pharmacological action. Thus, nanoemulsions demonstrate potential as a promising option for the dermal administration of essential oils [24,25].

Essential oils are a complex mixture, comprising a multitude of substances with diverse biological properties. This transforms them into a product with multi-target action, which can be enhanced by nanotechnology. The aim of the study was to develop, characterize, and assess the in vitro biological effects of a nanoemulsion containing the essential oil of *Melaleuca leucadendron* leaves for potential use in the prevention and topical treatment of melanoma.

## 2. Results and Discussion

### 2.1. Yield of Essential Oil Extraction

**Figure 1:**
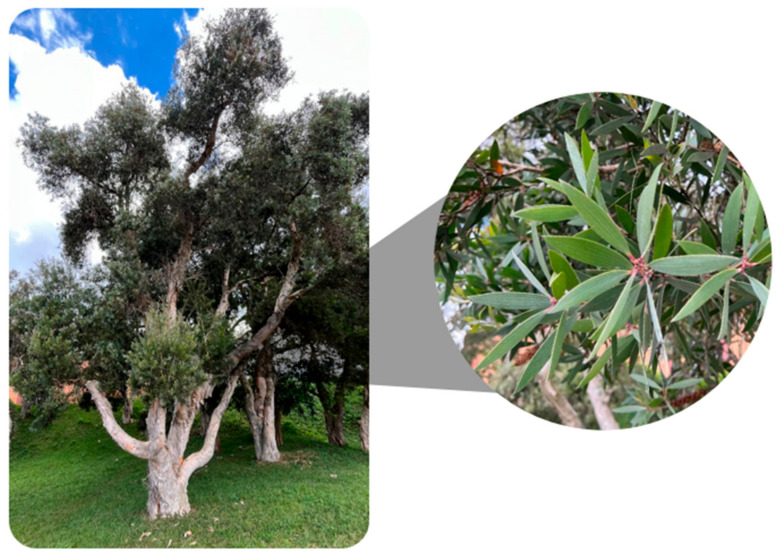
Photo of *Melaleuca leucadendron* plant cultivated at the Universidade Federal de Ouro Preto, Brazil (Photo by the authors).

By subtraction, we obtained 12.53 g of EO (13.5 mL) from 2.14 kg of *M. leucadendron* leaves (Figure 1), which is equivalent to 0.59% of oil extracted from the leaves.

In recent studies, researchers have undertaken the extraction of essential oil from *M. leucadendron*; however, limited attention has been given to reporting its extraction yield [26,27,28]. To our current understanding, the investigation conducted by Bautista-Silva et al. (2020) [29] yielded a similar result of 0.8%, aligning with our findings. In contrast, the research undertaken by Song et al. (2016) [30] documented an extraction yield of 1.75% for this essential oil. Notably, this value is approximately three times higher than the yield observed in our study. This variance could potentially be attributed to the disparities in the employed extraction methodologies as well as factors such as geographical location and collection timing [31]. It is plausible that these variables contributed to the observed differences in essential oil yield. In this context, seasonality studies could assist in identifying the optimal time of year for EO extraction, in addition to selecting other methods with greater productivity. Furthermore, different locations could even be indicated for *Melaleuca leucadendron* plantations.

### 2.2. Gas Chromatography–Mass Spectrometry (GC-MS) Analysis

**Figure 2:**
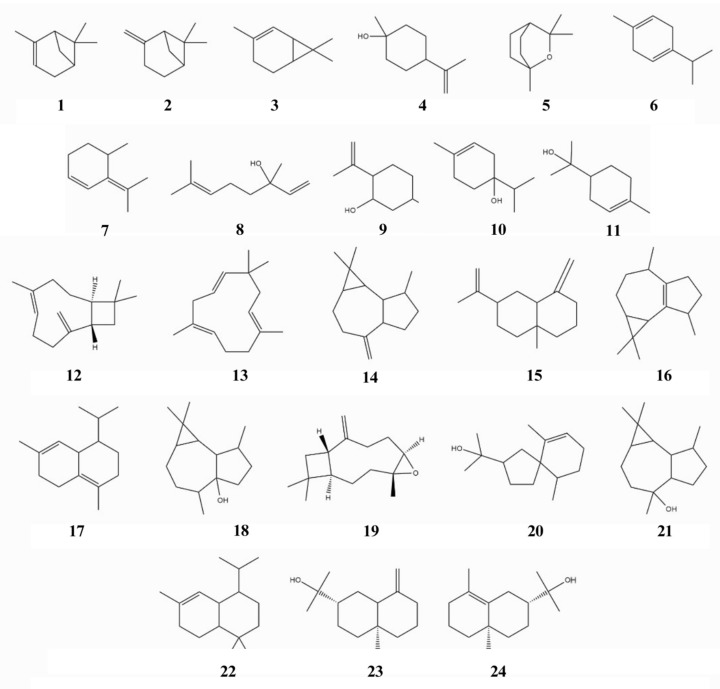
Major chemical constituents of essential oil from *Melaleuca leucadendron* leaves. (1) α-pinene; (2) β-pinene; (3)(+)-2-Carene; (4) β-terpineol; (5) Eucalyptol; (6) γ-terpinene; (7) Cyclohexene, 4-methyl-3-(1-methylethylidene)-; (8) Linalool; (9) Isopulegol; (10) Terpinen-4-ol; (11) α-terpineol; (12) Caryophyllene; (13) α-caryophyllene; (14) Aromadendrene; (15) β-selinene; (16) Ledene; (17) δ-cadinene; (18) Palustrol; (19) Caryophyllene epoxide; (20) Hinesol; (21) Viridiflorol; (22) α-cadinol; (23) β-selinenol; (24) Selinenol.

The essential oil obtained was chemically characterized by GC-MS (Figure S1, Supplementary Material). The presence of monoterpenes and sesquiterpenes was observed (Table 1 and Figure 2). These compounds offer insight into the rich chemical diversity and complexity of the constituents within the analyzed sample and likely contribute to the demonstrated activities of this essential oil, whether in isolation or through potential synergistic interactions with other constituents.

The essential oil obtained is composed of monoterpenes (43.76%)—with α-pinene (8.19%), β-terpineol (17.09%), and α-terpineol (6.65%) being the main ones—and sesquiterpenes (45.78%). The sesquiterpene hinesol stands out, being present in a high concentration (28.74%).

The study conducted by Bautista-Silva et al. (2020) [24] demonstrated that the essential oil derived from *M. leucadendron* leaves was predominantly composed of monoterpenoids (77.43%). Furthermore, the analysis highlighted four major compounds: α-pinene (9.06%), limonene (32.00%), 1,8-cineole (17.32%), and viridiforol (14.89%). Although some compounds may appear in similar or varying proportions, the unique composition of essential oils is notably influenced by factors such as climate, time of collection, diurnal variations, and the developmental stage of the plant. This reinforces the need for further studies that indicate ways to obtain standardized EO.

### 2.3. Nanoemulsion

#### 2.3.1. Zeta Potential, Hydrodynamic Diameter, Polydispersity Index and Centrifugation Test of the Nanoemulsions

The assessment of Zeta potential serves as a key indicator of particle nanoemulsion stability. When particles exhibit a significantly negative or positive Zeta potential, they tend to repel each other, preventing aggregation. Some researchers regard that a Zeta potential value exceeding |25| mV is favorable for stability [32,33]. The average Zeta potential value after analyzing all periods for NE-EO was −18.3 mV, while for NE-B, it was −19.1 mV. The Zeta potential values obtained from the formulated products were close but lower than the indications during all the time periods examined (Table 2).

Overall, there was no significant difference in the Zeta potential between NE-EO and NE-B during each period. However, there were statistical differences on days 7 and 28, with NE-EO showing greater stability on day 7 and NE-B showing greater stability 28 days after development. Additionally, the surface charge of NE-EO nanoparticles varied more between days compared to NE-B, which only showed a difference on day 7 after the development of the blank nanoemulsion. Despite this, the nanoemulsions remained stable after their development, which can be attributed to steric and electrostatic factors that contribute to stabilization in colloidal dispersions [34].

Dispersion systems characterized by droplet sizes falling within the nanometer scale (50–500 nm) are commonly referred to as nanoemulsions [23]. In our formulations, the droplet exhibited an average size after all analyzed periods of 182.7 nm (NE-EO) and 137.0 nm (NE-B), as quantified through photon correlation spectroscopy analysis. The hydrodynamic size of NE-EO was larger than that of NE-B, as expected due to the presence of essential oil in the internal phase. However, the average size of NE-EO is still small enough to be effective in topical applications, particularly in terms of cell penetration, which may contribute to its effectiveness against melanoma [35]. Furthermore, particle size distribution can assume two distinct states: polydisperse or monodisperse, showcasing broad or narrow distributions, respectively. Employing a scale from 0 to 1, a polydispersity index value < 0.3 is deemed favorable, suggesting a population of particles that exhibit a monodisperse system [36]. Hence, it can be concluded that the formulations possess a monodisperse profile, which is substantiated by their monomodal nanometer size distributions (Table 3).

The polydispersity index (PDI) of NE-EO after 1, 14, and 28 days increased after development compared to NE-B. During the evaluation period spanning from 1 to 28 days, noteworthy variations were absent in both particle size and PDI. Statistically significant differences were observed when comparing the same sample at different periods. The NE-B polydispersity index increased on days 21 and 28 after development. The constancy observed in the majority indicates a high degree of stability for the systems developed. This finding aligns with the outcomes of a study conducted by Seibert et al. (2018) [31], which demonstrated that nanoemulsions harboring the essential oil of *Cymbopogon densiflorus* leaves maintained their particle size and PDI even under stress conditions. This consistency further reinforces the potential stability of the nanoemulsions formulated in our study.

Finally, these findings are in alignment with the outcomes of the conducted centrifugation test. No evidence of phase separation or other indicators of nanoemulsion instability were observed. However, long-term stability studies must be carried out to define the optimal storage conditions and to prevent instability problems in a potential commercial product.

#### 2.3.2. Transmission Electronic Microscopy

Transmission electron microscopy (TEM) is an effective characterization technique that can provide data on the morphology and particle size of nanostructured systems [37,38].

**Figure 3:**
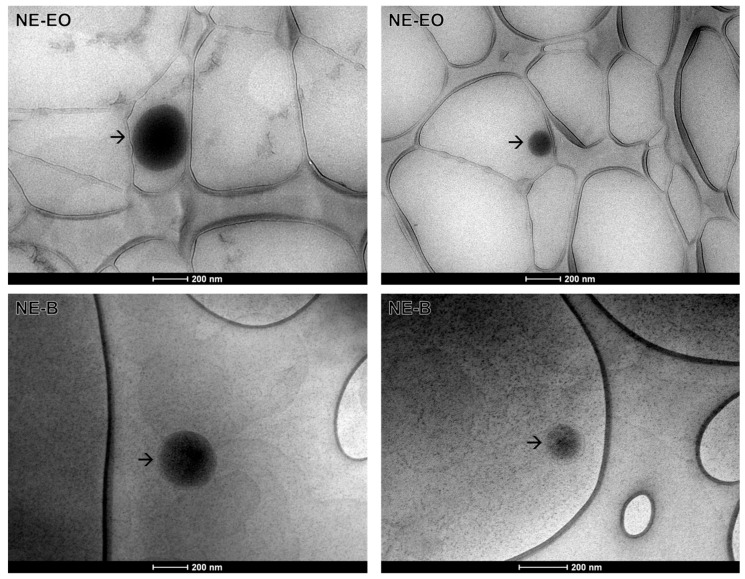
Morphology of nanoemulsions (NE-EO and NE-B) obtained by transmission electron microscopy at 43,000× magnification. The black arrows point to the nanoemulsion droplets. The white scale bars represent 200 nm.

In this context, an oval or spherical morphology is observed for NE-EO, while a spherical morphology is observed for NE-B, with a size close to 200 nm in both cases (Figure 3). This result is consistent with the hydrodynamic diameter results (Table 3).

#### 2.3.3. Rheological Behavior

The analysis of the formulation’s rheological behavior is important as this parameter affects the homogenization and stability of the final product over time. This analysis involves evaluating the material deformation and flow [39,40,41]. Furthermore, it is worth noting that certain sensory characteristics are influenced by rheology, such as spreadability, thickness, fluidity, and pourability [28]. This information is particularly relevant when formulating topical products (Table 4).

**Figure 4:**
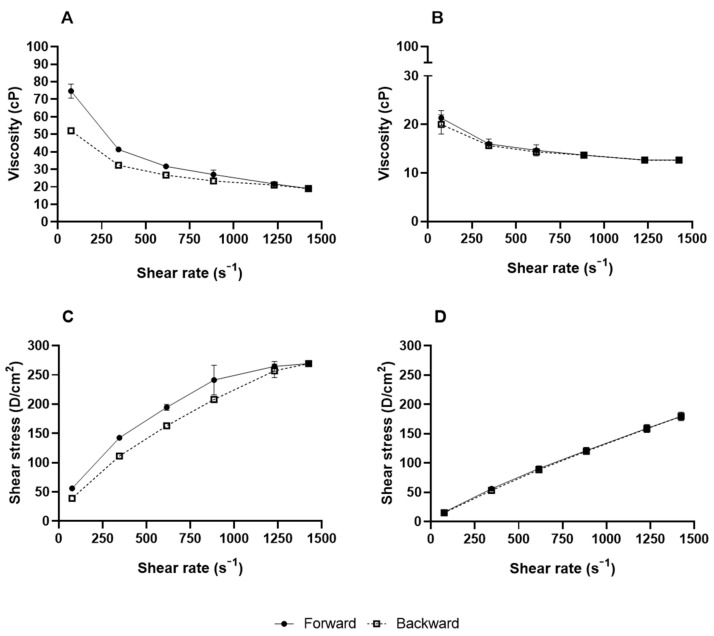
Representative steady shear flow curve of the NE-EO (**A**) and of the NE-B (**B**); representative rheogram of the NE-EO (**C**) and of the NE-B (**D**).

The flow index (n) reaches the unit value only for very low or very high deformation rates, indicating the transition to a more Newtonian behavior [42]. However, the results indicate that the flow rate was less than 1, revealing a pseudoplastic behavior in which the viscosity decreases with the increase in the shear rate (Figure 4A) and with the presence of thixotropy, in which the forward and backward curves did not coincide and formed a hysteresis loop for NE-EO (Figure 4C) [43]. This characteristic was observed in graphs fitted to the power-law model, which showed that subjecting the sample to agitation or shear caused temporary disruption of its particles or internal structures, facilitating the flow of the material. Nevertheless, when the agitation ceases, these structures require time to reconstitute and regain their original viscosity [44]. These characteristics are advantageous for a formulation because the decrease in apparent viscosity with increasing stress makes the system more fluid, thereby facilitating flow [45]. Additionally, pseudoplastic formulations can maintain an adequate initial viscosity to facilitate application and still thicken under rest, avoiding excessive dripping and ensuring proper adhesion to the skin [46]. This would be advantageous, as it would be possible to apply a thin layer of the product that is easy to spread, reducing the coarse and poor sensorial appearance presented by some pharmaceutical products for dermal application.

### 2.4. Photoprotective Activity

When evaluating melanoma prevention, in vitro photoprotection evaluation is an interesting technique to consider. This is because UV radiation is the primary cause of melanoma development [47,48]. The results of the in vitro photoprotective assay conducted in EO are presented in Table 5.

Observations underscore that EO, even at this maximum concentration, exhibited SPF unsatisfactory values since both the FDA (Food and Drug Administration of the United States) and the European Union advocate for SPF values exceeding 15.00 in pharmaceutical products meant for robust safeguarding against harmful UV radiation [49]. Scant literature reports touch upon the SPF of essential oil, likely attributed to their relatively subdued activity in this realm. To the best of our understanding, the study by Lohani; Mishra; Verma (2019) [50] stands alone in reporting SPF values of 6.45 for essential oil derived from *Pelargonium graveolens* leaves (0.1%) and SPF values of 8.36 for essential oil derived from *Calendula officinalis* flowers (0.1%). These findings diverge from the SPF values associated with the essential oil from leaves of *M. leucadendron*, probably due to differences in composition.

Despite the initially observed low activity of the EO, our investigation into the SPF of the developed nanoemulsions held significant importance. This inquiry was prompted due to this intended final application form (Table 6).

The SPF values of the diluted formulations may seem suboptimal at first glance. However, it is crucial to factor in the dilution effect, as these nanoemulsions are not intended for use in their diluted state. Upon accounting for this, the calculated SPF values surpassed the thresholds set by the FDA, indicating the potential efficacy of both NE-EO and NE-B. Particularly noteworthy is the effect observed with the presence of EO in the constitution, leading to SPF values approximately twice as high for NE-EO compared to NE-B. This outcome positions the NE-EO as a promising formulation. However, NE-B also presented a satisfactory SPF, which may be related to the presence of *Euterpe oleracea* vegetable oil in the formulation. Other researchers’ reports reinforce this assumption. In a study by Arianto; Cello; Bangun (2019) [51], a sunscreen nanoemulsion combining 3.00% avobenzone, 7.50% octyl methoxycinnamate, 2.73% soybean oil, and 0.27% liquid paraffin exhibited an SPF value of 21.57. This value surpassed those of the sunscreen nanoemulsion lacking soybean oil (SPF = 16.52) and the emulsion containing sunscreen alone (SPF = 15.10). Similarly, in research by Baccarin et al. (2015) [52], a nanoemulsion containing *Punica granatum* seed oil and the ethyl acetate fraction from the bark extract of the same plant species demonstrated an SPF close to 25.00. This SPF value exceeded that of another nanoemulsion containing the same fraction but formulated with medium-chain triglycerides. This finding underscores the contribution of oils, often enriched with long-chain triglycerides, in enhancing the SPF of nanoemulsions.

### 2.5. Antioxidant Activity

UV radiation possesses the capability to induce the formation of free radicals within the skin, thus potentially fostering the emergence of diverse skin cancer types, including melanoma [53]. In this context, the integration of antioxidant compounds, primarily originating from botanical sources, holds the potential not only to limit oxidative damage to the skin but also to curb the reliance on synthetic components in photoprotective products, given their potential adverse effects on human health [16,54]. The results of the antioxidant activity of the EO, NE-EO, and NE-B are shown in Table 7.

Other essential oils have already been demonstrated to possess potent antioxidant properties, such as the essential oil of *Syzygium aromaticum*, a plant of Indian origin known as clove. A number of studies have already reported the effect of this essential oil, and its activity of eliminating 50% of radicals varies between 32.55 and 100.65 µg/mL for the method using DPPH [55,56,57] and between 8.50 and 78.98 µg/mL using the ABTS radical [58,59,60].

Eugenol, a major constituent of *S. aromaticum* essential oil, has been identified as a strong antioxidant and has been used as a positive control (C+) in our study. As anticipated, the essential oil (EO) demonstrated an antioxidant effect, although to a lesser extent than eugenol. This is due to the fact that eugenol is a pure aromatic substance capable of eliminating radicals and stabilizing itself due to the presence of the hydroxyl group and conjugations in its chemical structure [61]. The antioxidant properties of EO result from a complex interaction of several compounds, each with its unique level of activity.

Previous research has documented that the terpenes in its composition have antioxidant effects, as demonstrated by various radical scavenging assays [62,63]. Monoterpenes, including α-pinene, γ-terpinene, terpinene-4-ol, and α-terpineol, have already been identified as compounds present in *Melaleuca alternifolia* and also make up *Melaleuca leucadendron*. In this context, a comparison of the antioxidant activity of *M. alternifolia* with those of our study reveals that the inhibition of 50% of DPPH radicals occurs between 12.50 and 48.35 µg/mL, while that of ABTS occurs between less than 3.12 and 38.99 µg/mL [64,65,66]. Although they share certain compounds, the relative proportions of these compounds between species vary. Furthermore, each species possesses specific compounds that contribute to the observed differences in activity.

It was not possible to determine specific IC_50_ values for the DPPH method for the evaluation of antioxidant activity in nanoemulsions. However, this was possible using the ABTS method. Previous studies have shown that the ABTS radical method is more versatile than the DPPH method [31]. This is because the ABTS model can evaluate the radical scavenging activity of both polar and nonpolar samples. Additionally, the working solution is soluble in aqueous and organic solvents over a wide range of pH values. Furthermore, the reaction time is shorter than the DPPH assay. The results of the ABTS method showed greater sensitivity for the antioxidant properties of the samples compared to the DPPH method. The IC_50_ of NE-EO was found to be 265.13 µg/mL ± 12.94, which is lower than that of NE-B (408.33 µg/mL ± 13.37), indicating a stronger intrinsic antioxidant effect of EO in this formulation.

### 2.6. Cytotoxicity

Cytotoxicity assessments serve a dual purpose: they provide insights into safe dosage levels for diverse applications and identify effective dosages against tumor cells [67,68,69]. In this study, our focus was on elucidating the impact of the developed nanoemulsions on non-tumorigenic cells, specifically L-929 fibroblasts and NGM human melanocytes, as well as on tumorigenic cells (B16-F10 melanoma and MeWo human melanoma), since essential oils are being explored as a potential alternative treatment for cancer due to their promising biological activities in preventing tumorigenesis and the progression of different tumors, including melanoma [19]. The results of the cell viability assessments (Table 8) indicate that the EO and NE-EO have more cytotoxic effects on melanoma cells than on non-tumoral cells.

The results show that NE-EO enhanced the cytotoxic effects of EO against L-929, B16-F10, NGM, and MeWo cells 24 and 48 h after treatment. Notably, the SI also increased, indicating an improved antitumor effect of the nanoemulsified EO against murine cells. The greater effect of NE-EO compared to EO may be attributed to enhanced membrane permeability conditions resulting from the use of nanoemulsion. The characteristics of surface charge and nanometric size and the greater solubility of active constituents facilitate superior activity against a wider range of cancer cells, which are rapidly killed following initial exposure.

While there are currently no reports on the anti-melanoma effect of EO, other studies suggest its potential. Previous studies have reported that the essential oil of *Melaleuca alternifolia*, when combined with dabrafenib and/or trametinib, can synergistically reduce the viability of melanoma cells by activating apoptosis. This activity has been attributed to the compounds α-terpineol, terpinolene, and terpinen-4-ol. The latter has antitumor and pro-apoptotic effects and is present in the EO [19]. Other compounds identified by chemical characterization of the present essential oil, hinesol and α-pinene, showed cytotoxic activity that has already been reported against tumor cell lines [20,21]. Therefore, these can be considered the likely bioactives of EO.

When comparing our results with those in the literature regarding the cytotoxicity of nanoemulsions against non-tumor cells, we observe consistency. Our results are in line with previous findings that report the absence of cytotoxicity against L-929 fibroblasts of astaxanthin-loaded nanoemulsion and its blank nanoemulsion produced using the phase inversion method up to doses of 500 μg/mL [70]. Previous research investigating nanoemulsions with particle sizes ranging from 100 to 200 nm, incorporating substances such as docetaxel and curcumin, reported a lack of cytotoxicity for formulations falling within the 150 to 200 nm size range when exposed to MRC-5 cells over a 48-h period, even at higher concentrations [71]. Similarly, another investigation involving nanoemulsions containing various red raspberry seed oils, formulated via the phase inversion method, exhibited a favorable safety profile when tested against MRC-5 cells [72]. These studies align with the outcomes of our present study, thus further substantiating the potential for secure topical application of our developed nanoemulsion.

A comparison of the cytotoxicity results against melanoma with those of other studies reveals that the research conducted by [73] exhibited that a nanoemulsion incorporating coffee oil and algal oil effectively hindered the growth of B16-F10 melanoma cells while leaving the viability of human CCD986SK non-tumor fibroblasts unaffected. In another study, a nanoemulsion containing *E. oleracea* oil was found to have potent photosensitization potential for photodynamic therapy, both in vitro and in vivo. Photodynamic therapy, in conjunction with an *E. oleracea* oil-infused nanoemulsion, induced an 85% cell death rate in B16-F10 cells while preserving the viability of non-tumor cells [25]. The alignment of effects suggests that the observed effect in our study may also be attributed to *E. oleracea* oil, indicating a potential interaction of effects between the two oils.

Finally, an interesting study demonstrated the effectiveness of reformulating dacarbazine for topical application, resulting in significant inhibition of the growth of melanoma. In a mouse xenotransplantation model, the topical application of a dacarbazine nanoemulsion showed up to 10-fold greater percentage reductions in tumor size compared to a dacarbazine suspension preparation [74]. These results suggest that less-invasive therapeutic approaches with nanotechnology, such as the topical application of antitumor agents, deserve greater attention and future development.

## 3. Materials and Methods

### 3.1. Plant Material

*M. leucadendron* leaves were collected in Ouro Preto, MG, Brazil (20°23′48.8″ S, 43°30′32.5″ W), in June 2023. A voucher specimen (OUPR 41732) was deposited in the Herbarium of Professor José Badini at Universidade Federal de Ouro Preto, MG, Brazil. The research was registered for access (approval: A2CC35F) in the Sistema Nacional de Gestão do Patrimônio Genético e do Conhecimento Tradicional Associado (SisGen).

### 3.2. Extraction of the Essential oil from Melaleuca leucadendron Leaves

A total of 2.14 kg of *M. leucadendron* leaves were carefully harvested and thoroughly washed before being cut. Then, hydrodistillation was performed using a Linax^®^ apparatus for approximately 3 h. After completing the distillation process, the obtained EO was collected with micropipettes (Eppendorf, São Paulo, Brazil) and then precisely transferred into a tube. The tube underwent centrifugation (Kasvi, São José dos Pinhais, Brazil) at 4000 rpm for 10 min to eliminate water traces. Post-centrifugation, a secondary collection step was performed, and the oil was transferred to a sterilized container for preservation. The mass of the essential oil was quantified by subtracting the post-extraction weight of the container from the initial weight. The essential oil yield was determined by comparing the weight of the extracted essential oil with the initial mass of the harvested leaves, which was established as the baseline (100%).

### 3.3. Gas Chromatography–Mass Spectrometry (GC-MS) Analysis

The chemical composition analysis of the EO was performed using a gas chromatography–mass spectrometer (GCMS-QP2020 NX Shimadzu, Kioto, Japan) equipped with a fused silica capillary column (ZB-5MSi 30 m × 0.25 mm × 0.25 μm, RESTEK, Centre County, PA, USA). To quantitatively assess compound concentrations, the relative percentages (% content) of all components were calculated by integrating their respective peak areas in the total ion chromatogram (TIC, *m*/*z* 50–600). Sample preparation involved combining 500 μL of essential oil with 500 μL of dichloromethane (Êxodo Científica, São Paulo, Brazil), followed by injection under the following conditions: helium (99.999%) (White Martins, Belo Horizonte, Brazil) as the carrier gas at a constant flow of 1.1 mL min^−1^; an injection volume of 1 μL; an injector split ratio of 1:10; injector temperature of 280 °C; electron impact mode at 70 eV; ion-source temperature of 280 °C; and a transfer line temperature of 280 °C. The oven temperature was programmed to increase from 60 °C to 200 °C in 10 min and from 200 °C to 280 °C in 3 min. The identification of essential oil constituents was accomplished by interpreting spectra obtained from a database (Library:NIST05s), and compounds with content above 0.5% were reported.

### 3.4. Nanoemulsion Development

The nanoemulsions were created through the phase inversion emulsification method [26]. The composition of NE-EO consisted of EO (2% *v*/*v*), *Euterpe olereacea* oil (Gran Oils Brazil, Santo André, SP, Brazil) (5% *v*/*v*) as the oil phase, ultrapure water (Milli-Q, Millipore, Merck Millipore, São Paulo, Brazil) (88% *v*/*v*) as the aqueous phase, and nonionic surfactants BRIJ S2 (Polyoxyethylene fatty ether, Croda, Brazil) (2% *w*/*w*) and RH 400 (PEG-40 Hydrogenated Castor Oil, OXITENO, São Paulo, Brazil) (3% *w*/*w*). The composition of NE-B consisted of *E. oleracea* oil (Gran Oils) (5% *v*/*v*) as the oil phase, ultrapure water (90% *v*/*v*) as the aqueous phase, and nonionic surfactants BRIJ S2 (Polyoxyethylene fatty ether, Croda, Brazil) (2% *w*/*w*) and RH 400 (PEG-40 Hydrogenated Castor Oil, OXITENO, Brazil) (3% *w*/*w*). The oil phase and surfactants were heated to 75 ± 2 °C. The water phase was also heated to the same temperature. The two phases reached the mentioned temperature, the EO was added, and the water was gradually added to the oil phase while agitating for 1 min at 600 rpm using a mechanical stirrer (Fisaton, model 713D, São Paulo, Brazil). After 1 min, the emulsions were transferred to the magnetic agitator (heating magnetic stirrer) (Fisaton, São Paulo, Brazil) until it completely cooled down. After 24 h of nanoemulsion preparation, macroscopic and microscopic analyses were conducted to assess organoleptic attributes and homogeneity. The blank nanoemulsion was prepared in the same manner but without the presence of EO.

#### 3.4.1. Zeta Potential, Hydrodynamic Diameter and Polydispersity Index

The physicochemical properties of the sample were analyzed employing the Zetasizer^®^ Nano Series (Malvern, UK) to determine the Zeta potential, particle hydrodynamic diameter, and polydispersity index. Zeta potential was determined through electrophoretic mobility measurements of suspended particles, where samples were directly placed in a capillary cell. The average size (hydrodynamic diameter) and polydispersity index (PDI) were assessed using photon correlation spectroscopy. To determine the particle size at room temperature, 20 μL of the nanoemulsion was diluted in 1980 μL of ultrapure water. The laser incidence angle on the sample was 90°. All measurements were performed in triplicate 1, 7, 14, 21, and 28 days after developing the nanoemulsions.

#### 3.4.2. Centrifugation Test

For the centrifugation procedure, 2000 μL of the nanoemulsions (prepared 1 day prior) was spun at 5000 rpm for 5 min. The experiment was repeated three times. Subsequently, the samples were observed for the presence or absence of phase separation.

#### 3.4.3. Transmission Electronic Microscopy

Lacey carbon screens, 300 mesh copper SEM^®^, were subjected to the glow discharge process in an argon atmosphere for one minute at a current of 10 kV, using the Bal-Tec MED20 metallizer (BalTec, Pfäffikon, Switzerland). Following the glow discharge, 3 µL of the sample was deposited on the screen, and one minute later, the excess sample was removed using filter paper. Subsequently, 3 µL of contrast agent (2% uranyl acetate) was deposited on the grid. Following a 30 s interval, the excess was removed with filter paper, and the grid was stored in the gridbox until analysis (which typically occurs within 24 h). The analysis was conducted using a transmission electron microscope (TEM)—Tecnai G2-12 Spirit Biotwin 120 kV (Thermo Fisher/FEI, Waltham, MA, USA) —at a voltage of 60 kV. The images were obtained using a charge-coupled device (CCD) camera at a magnification of 43,000×.

#### 3.4.4. Rheological Analysis

The rheological behavior of nanoemulsions was assessed using a Brookfield rheometer model DV-III cone-and-plate system, operated with RHEOCALC 3.0 software (AMETEK Brookfield, Middleborough, MA, USA). Rheological parameters were determined at 25 °C, employing the CP 40 spindle along with 500 μL of the sample. Measurements were taken 1, 7, 14, 21, and 28 days after preparation. The rotational speed was in a range of 10, 46, 82, 118, 164, and 190 rpm for ascending curve measurements. The descending curve was acquired by equally decreasing this rotational speed in intervals [26].

The analysis of the rheological behavior of the nanoemulsions utilized the power law, as expressed by the equation:

τ = k·γ^n^

In this equation, τ represents the shear stress, k denotes the consistency index, γ stands for the shear rate, and n signifies the flow index. This equation allows for the determination of the rheological behavior of the formulations.

### 3.5. Photoprotective Activity

The absorption readings of the EO, NE-EO, and NE-B were taken using the Genesys 10S UV-Vis Spectrophotometer (Thermo Fisher, Waltham, MA, EUA) to determine the maximum absorbance in the ultraviolet regions. To analyze the EO, solutions were prepared at concentrations from 100 to 500 μg/mL using dichloromethane P.A as solvent. To analyze NE-EO and NE-B, dilutions of 1% to 3% *v/v* were performed using 95% ethyl alcohol (Hexis Científica, Jundiaí, Brazil) as solvent. Scanning was performed in triplicate between the wavelengths of 200 to 800 nm in the UV-Vis Spectrophotometer with all sample solutions, using a quartz cuvette with an optical path of 1.0 cm, and the solvent of each solution is white in the respective reading. Through the equation of the Mansur method [75,76], it was possible to determine the value of the sun protection factor (SPF) of each concentration of the samples. The absorption readings used to calculate the SPF are between 290 and 320 nm, with intervals of 5 nm. The results obtained were examined according to the mean ± standard deviation of the mean percentage of reduction and/or increase in the sun protection factor.

### 3.6. Antioxidant Activity

#### 3.6.1. 2,2-Diphenyl-1-picrylhydrazyl (DPPH) Radical Inhibition

The antioxidant activity was assessed using an in vitro photocolorimetric method involving the radical DPPH, with adaptations [77]. Initially, one DPPH (Sigma-Aldrich, Saint Louis, MO, USA) solution was prepared at a concentration of 60 μg/mL in ethanol (Hexis Científica, Jundiaí, Brazil). Stock solutions of EO (solubilized in ethanol + 10% Tween 80), NE-EO, NE-B (both solubilized in ethanol), and the positive control (eugenol, solubilized in ethanol + 10% Tween 80) were prepared at different concentrations. Subsequently, the samples were arranged into 96-well microplates across six concentrations, yielding final concentrations following DPPH addition of 61.04, 122.07, 244.14, 488.28, 976.57 and 1953.13 μg/mL for the EO; 78.12, 156.25, 312.50, 625.00, 1250.00, 2500.00, 5000.00, and 10,000.00 μg/mL for the NE-EO and NE-B; and 0.16, 0.33, 0.65, 1.31, 2.62 and 5.24 µg/mL for the eugenol. After a 30 min incubation, absorbance was measured at 490 nm using a microplate reader (Thermo Fisher, Waltham, MA, USA). The assay was conducted in quadruplicate. The concentration required to inhibit 50% of the DPPH radicals (IC_50_) was determined using the percentage inhibition results, and outcomes were presented as the mean ± standard deviation of the IC_50_ values determined through non-linear regression using GraphPad Prism 8.0.1 software.

#### 3.6.2. 2,2′-Azinobis(3-ethylbenzothiazoline-6-sulfonic Acid) (ABTS) Radical Inhibition

The ABTS method was performed following Li et al. (2009) [78], with modifications. Stock solutions of EO (solubilized in ethanol + 10% Tween 80), NE-EO, NE-B (both solubilized in ethanol), and the positive control (eugenol, solubilized in ethanol + 10% Tween 80) were prepared at different concentrations. Then, 60 μL of these solutions was transferred to 96-well plates, in order to obtain final concentrations of 12.21, 24.71, 48.83, 97.66, 195.32 and 390.63 μg/mL for the EO; 25.19, 50.37, 100.74, 201.49, 402.97 and 805.94 for NE-EO; 26.95, 53.91, 107.81, 215.63, 431.25 and 862.50 μg/mL for NE-B; and 0.03, 0.07, 0.13, 0.26, 0.53 and 1.05 μg/mL for eugenol. After adding the samples, 240 μL of ABTS (Sigma-Aldrich, Saint Louis, MO, USA) was added, and the plate was incubated for 6 min, protected from light. Absorbance readings were taken on a microplate reader at 650 nm. All analyses were performed in quadruplicate. The concentration required to inhibit 50% of the ABTS radicals (IC_50_) was determined using the percentage inhibition results. This determination was carried out using GraphPad Prism^®^ 8.0.1, with non-linear regression. The results were presented as mean ± standard deviation.

### 3.7. Cytotoxicity Assay

#### 3.7.1. Cell Culture

The subcutaneous connective-tissue mouse fibroblast cell line (NCTC clone 929 [L cell, L-929, derivative of Strain L], ATCC CCL-1) and the murine metastatic melanoma cell line (B16-F10, ATCC CRL-6475) were grown in a basal culture medium containing RPMI Sigma-Aldrich^®^, supplemented with 10% FBS (Fetal Bovine Serum) (Gibco^®^, Waltham, MA, USA) and gentamicin (Thermo Fisher Scientific^®^, Waltham, MA, USA). Cells were incubated in a humidified atmosphere of 5% CO_2_ at 37 °C. Non-tumoral human melanocytes (NGMs) obtained from Banco de Células do Rio de Janeiro (BCRJ, Rio de Janeiro, RJ, Brazil), catalog number: 0190, were maintained and cultured in Dulbecco’s Modified Eagle Medium DMEM (4500 mg/L glucose)/HAM F12 (1:1) (Thermo Fisher Scientific, MA, USA) supplemented with 20% FBS, 10 mM HEPES, 2.5 mM L-glutamine, 0.5 mM sodium pyruvate, and 2.5 mM non-essential amino acids. The human melanoma cell line (MeWo (ATCC HTB-65)) was cultured in a complete growth medium that contained DMEM (4500 mg/L glucose) supplemented with 10% FBS, 10 mM HEPES, 2.5 mM L-glutamine, 0.5 mM sodium pyruvate, and 2.5 mM non-essential amino acids.

#### 3.7.2. Sulforhodamine B Method

Cell viability was evaluated using the sulforhodamine B assay (SRB) [67,69]. Cells were treated with samples dissolved in RPMI + 2% DMSO for EO and RPMI for NE-EO and NE-B, across six concentrations for L-929 and B16-F10, yielding final concentrations of 7.81, 15.63, 31.25, 62.50, 125.00, and 250.00 μg/mL for the EO and 390.63, 781.25, 1562.50, 3125.00, 6250.00, and 12,500.00 μg/mL for the NE-EO and NE-B. NGM and MeWo cells were treated across eight concentrations, yielding final concentrations of 7.81, 15.63, 31.25, 62.50, 125.00, 250.00, 500.00, and 1000.00 μg/mL for the EO and 97.66, 195.31, 390.63, 781.25, 1562.50, 3125.00, 6250.00, and 12,500.00 μg/mL for the NE-EO and NE-B. The cells incubated in the absence of the test sample and in the presence of 2% DMSO were used as a negative control. After 24 and 48 h of incubation, the medium was removed and cells were fixed with cold 20% trichloroacetic acid for 1 h at 4 °C. The microtiter plate was washed with distilled water and dried. Thereafter, fixed cells were stained for 30 min with 0.1% SRB dissolved in 1% acetic acid. The plate was washed again with 1% acetic acid and allowed to dry, and 200 µL of 10 mmol/L TRIS buffer (pH 10.5) was added to sustain solubilization at room temperature for ~30 min. Sample absorbance was read in the spectrophotometer (540 nm), and CC_50_ was calculated by the non-linear regression of normalized data using GraphPad Prism 8.0.1 software. The results were expressed as the mean ± standard deviation of triplicates.

#### 3.7.3. Selectivity Index

The selectivity index (SI) was calculated as the ratio of CC_50_ L-929 to CC_50_ B16-F10 and as the ratio of CC_50_ NGM to CC_50_ MeWo.

### 3.8. Statistical Analysis

The normal distribution of the presented data was verified using the Shapiro–Wilk test. Differences between NE-EO and NE-B, as well as between 1, 7, 14, 21, and 28 days after the development of each nanoemulsion, were analyzed using the T-test for Zeta potential, hydrodynamic size, polydispersity index, and rheological analysis. A significance threshold of *p* < 0.05 was considered for determining statistically significant differences. The GraphPad Prism 8.0.1 software was utilized.

## 4. Conclusions

Plants are a rich source of compounds with great potential for use in cosmetics and pharmaceuticals. The proposal for an O/W nanoemulsion containing the essential oil of *Melaleuca leucadendron leaves* is a promising one. It has good stability and a pseudoplastic profile, which means it could adhere well to the skin. The external, aqueous phase of the nanoemulsion would allow it to be spreadable on the skin, and at the same time, a hydrophobic film of its internal, oily phase would be formed after a certain time of application, which could be beneficial in promoting a photoprotective and antioxidant effect. Taking into account the versatility of this type of formulation, such as its amphiphilic nature, reduced particle size, and surface charge, an antitumor effect against already established melanoma could be facilitated. Thus, this nanoemulsion not only emerges as a possible photoprotector and antioxidant but also as a viable adjuvant in the treatment of melanoma. In order to use the nanoemulsion for preventive purposes, studies to evaluate its association with synthetic chemical or physical filters are encouraged, as well as those evaluating water resistance. Additionally, polymers that do not alter the physicochemical conditions of the nanoemulsion can be added. In order to use it for the treatment of melanoma, in vivo and clinical studies are necessary to fully evaluate the efficacy and safety of the developed nanoemulsion.

## Figures and Tables

**Table 1:** Major chemical constituents of essential oil from *Melaleuca leucadendron* leaves.

No.	RT (min)	Compound	Class	Area (%)	Similarity
1	5.78	α-pinene	Monoterpene	8.19	95
2	6.84	β-pinene	Monoterpene	2.96	95
3	8.02	(+)-2-Carene	Monoterpene	0.51	93
4	8.60	β-terpineol	Monoterpene	17.09	89
5	8.66	Eucalyptol	Monoterpene	2.88	89
6	9.34	γ-terpinene	Monoterpene	1.43	95
7	10.29	Cyclohexene, 4-methyl-3-(1-methylethylidene)-	Monoterpene	0.60	93
8	10.68	Linalool	Monoterpene	1.03	95
9	12.27	Isopulegol	Monoterpene	0.54	96
10	13.42	Terpinen-4-ol	Monoterpene	1.88	94
11	14.02	α-terpineol	Monoterpene	6.65	94
12	21.80	Caryophyllene	Sesquiterpene	2.22	94
13	22.90	α-caryophyllene	Sesquiterpene	0.62	95
14	23.14	Aromadendrene	Sesquiterpene	0.74	94
15	23.97	β-selinene	Sesquiterpene	0.83	94
16	24.26	Ledene	Sesquiterpene	1.33	91
17	25.10	δ-cadinene	Sesquiterpene	0.60	90
18	26.55	Palustrol	Sesquiterpene	1.05	94
19	27.04	Caryophyllene epoxide	Sesquiterpene	2.23	93
20	27.83	Hinesol	Sesquiterpene	28.74	88
21	27.97	Viridiflorol	Sesquiterpene	4.43	92
22	28.81	α-cadinol	Sesquiterpene	1.00	90
23	29.12	β-selinenol	Sesquiterpene	0.76	92
24	29.21	Selinenol	Sesquiterpene	1.23	85

RT: retention time.

**Table 2:** Zeta potential of the nanoemulsions according to the analyzed period. Results expressed as mean ± standard deviation.

Period (Days)	Zeta Potential (mV)	
	NE-EO	NE-B
1	−18.0 ± 1.06 ^aA^	−19.9 ± 1.78 ^aA^
7	−23.0 ± 0.92 ^bA^	−15.6 ± 1.50 ^bB^
14	−15.1 ± 0.60 ^cA^	−19.1 ± 1.83 ^aA^
21	−20.2 ± 0.30 ^aA^	−21.4 ± 1.69 ^aA^
28	−15.4 ± 0.30 ^cA^	−19.5 ± 0.62 ^aB^

Different lowercase letters mean a significant statistical difference between the same sample in different periods (*p* < 0.05); Different capital letters mean a statistically significant difference between NE-EO and NE-B in the same period (*p* < 0.05).

**Table 3:** Particle size values and polydispersity index of the nanoemulsions according to the analyzed period. Results expressed as mean ± standard deviation.

Period (Days)	Size (nm)		PDI	
	NE-EO	NE-B	NE-EO	NE-B
1	179.5 ± 1.852 ^aA^	138.2 ± 0.833 ^aB^	0.23 ± 0.010 ^aA^	0.15 ± 0.011 ^aB^
7	184.0 ± 2.676 ^aA^	137.6 ± 0.954 ^aB^	0.23 ± 0.031 ^aA^	0.17 ± 0.004 ^aA^
14	181.1 ± 0.950 ^aA^	135.6 ± 0.458 ^aB^	0.25 ± 0.029 ^aA^	0.17 ± 0.011 ^aB^
21	188.6 ± 14.330 ^aA^	136.1 ± 0.208 ^aB^	0.27 ± 0.048 ^aA^	0.18 ± 0.005 ^bA^
28	180.2 ± 1.305 ^aA^	137.3 ± 1.185 ^aB^	0.25 ± 0.014 ^aA^	0.18 ± 0.004 ^bB^

Different lowercase letters mean a significant statistical difference between the same sample in different periods (*p* < 0.05); Different capital letters mean statistically significant difference between NE-EO and NE-B in the same period (*p* < 0.05).

**Table 4:** Rheological behavior parameters of the NE-EO and NE-B according to the time period analyzed. Results expressed as mean ± standard deviation.

Period (Days)	Consistency Index (cP)		Flow Rate (n)		Confidence (%)	
	NE-EO	NE-B	NE-EO	NE-B	NE-EO	NE-B
1	240.70 ± 13.308 ^aA^	12.63 ± 1.115 ^aB^	0.60 ± 0.006 ^aA^	0.86 ± 0.015 ^aB^	92.6 ± 0.808	97.1 ± 1.015
7	259.66 ± 28.854 ^aA^	18.90 ± 0.916 ^bB^	0.60 ± 0.012 ^aA^	0.87 ± 0.030 ^aB^	96.7 ± 0.404	97.7 ± 0.305
14	322.40 ± 12.322 ^bA^	25.33 ± 1.436 ^cB^	0.61 ± 0.006 ^aA^	0.87 ± 0.010 ^aB^	92.4 ± 0.404	97.1 ± 0.289
21	343.33 ± 39.172 ^bA^	42.23 ± 1.616 ^dB^	0.61 ± 0.026 ^aA^	0.83 ± 0.006 ^aB^	90.5 ± 1.137	98.0 ± 0.737
28	268.20 ± 5.415 ^aA^	85.00 ± 32.568 ^eB^	0.64 ± 0.020 ^aA^	0.75 ± 0,052 ^bA^	89.6 ± 0.472	97.3 ± 0.557

Different lowercase letters mean a significant statistical difference between the same sample in different periods (*p* < 0.05); Different capital letters mean statistically significant difference between NE-EO and NE-B in the same period (*p* < 0.05).

**Table 5:** SPF of EO in dichloromethane at concentrations from 100 to 500 μg/mL. Results represent the mean ± SD of triplicates of the experiments.

Concentration	Solar Protection Factor (SPF)
	EO
100 μg/mL	0.1350 ± 0.0273
200 μg/mL	0.1964 ± 0.0144
300 μg/mL	0.2658 ± 0.0125
400 μg/mL	0.3423 ± 0.0265
500 μg/mL	0.4213 ± 0.0432

**Table 6:** SPF of NE-EO and NE-B in 95% ethyl alcohol at concentrations from 1% to 3% *v*/*v*. Results represent the mean ± SD of triplicates.

Concentration	Solar Protection Factor (SPF)	
	NE-EO	NE-B
1% *v*/*v*	0.2892 (28.92 ^A^) ± 0.0039	0.1194 (11.94 ^A^) ± 0.0019
2% *v*/*v*	0.6334 (31.67 ^B^) ± 0.0025	0.2421 (12.10 ^B^) ± 0.0384
3% *v*/*v*	0.9532 (30.82 ^C^) ± 0.0236	0.4946 (15.99 ^C^) ± 0.0097

A: value corrected by the dilution factor (100.00); B: value corrected by the dilution factor (50.00); C: value corrected by the dilution factor (32.33).

**Table 7:** Antioxidant activity of EO, NE-EO and NE-B. Results represent the mean ± SD of triplicates of the experiments.

Sample	DPPH	ABTS
	IC_50_ (µg/mL)	IC_50_ (µg/mL)
EO	277.38 ± 22.12	40.72 ± 5.70
NE-EO	>10,000.00 (>200.00 ^A^)	265.13 (5.30 ^A^) ± 12.94
NE-B	>10,000.00	408.33 ± 13.37
C+	1.17 ± 0.28	0.24 ± 0.02

IC_50_: Concentration required to inhibit 50% of radicals; C+: eugenol; A: IC_50_ corrected according to the EO concentration in the nanoemulsion.

**Table 8:** Cytotoxicity of EO, NE-EO, and NE-B against L-929, B16-F10, NGM, and MeWo cells 24 and 48 h after treatments.

Skin Cell Line		Sample		
		EO	NE-EO	NE-B
L-929 CC_50_ (µg/mL)	24 h	81.10 ± 4.42	2124.33 (42.49 ^C^) ± 433.21	3311.00 ± 429.85
	48 h	63.40 ± 1.85	846.33 (16.93 ^C^) ± 112.29	2030.10 ± 177.66
B16-F10 CC_50_ (µg/mL)	24 h	60.61 ± 2.16	945.30 (18.91 ^C^) ± 82.14	3008.67 ± 40.08
	48 h	44.64 ± 2.00	566.87 (11.34 ^C^) ± 31.03	2003.67 ± 111.38
SI ^A^	24 h	1.34	2.25	1.10
	48 h	1.42	1.49	1.01
NGM CC_50_ (µg/mL)	24 h	155.2 ± 29.15	1067.0 (21.34 ^C^) ± 24.23	2091.0 ± 66.19
	48 h	129.6 ± 27.78	508.5 (10.17 ^C^) ± 13.31	1406.0 ± 25.87
MeWo CC_50_ (µg/mL)	24 h	58.5 ± 13.03	404.1 (8.10 ^C^) ± 11.63	1027.0 ± 11.43
	48 h	50.46 ± 19.33	391.71 (7.83 ^C^) ± 13.75	919.8 ± 13.86
SI ^B^	24 h	2.65	2.64	2.04
	48 h	2.57	1.30	1.53

CC_50_: cytotoxic concentration for 50% of cells; SI: selectivity index; A: CC_50_L-929/CC_50_B16-F10; B: CC_50_NGM/CC_50_MeWo; C: CC_50_ corrected according to the EO concentration in the nanoemulsion.

## Data Availability

All data generated or analyzed during this study are included in this published article.

## Supplementary Materials

The following supporting information can be downloaded at: https://www.mdpi.com/article/10.3390/ph17060721/s1, Figure S1: Chromatogram of the major chemical constituents of the essential oil extracted from *Melaleuca leucadendron* leaves.

## Abbreviations

UV	Ultraviolet
NE-EO	Nanoemulsion prepared with essential oil
NE-B	Nanoemulsion prepared without essential oil
EO	Essential oil of *Melaleuca leucadendron* leaves
DNA	Deoxyribonucleic acid
GC-MS	Gas chromatography–mass spectrometry
cP	Consistency index
n	Flow rate
SPF	Solar protection factor
FDA	Food and Drug Administration of the United States
C+	Positive control
SisGen	Sistema Nacional de Gestão do Patrimônio Genético e do Conhecimento Tradicional Associado
TIC	Total ion chromatogram
PDI	Polydispersity index
DPPH	2,2-diphenyl-1-picrylhydrazyl
ABTS	2,2′-Azinobis(3-ethylbenzothiazoline-6-sulfonic acid)
ATCC	American Type Culture Collection
BCRJ	Banco de Células do Rio de Janeiro
FBS	Fetal Bovine Serum
SRB	Sulforhodamine B
TEM	Transmission electron microscopy
TIC	Total ion chromatogram
CCD	Charge-coupled device
