# Supplementary material for: Nanoemulsified Essential Oil of Melaleuca leucadendron Leaves for Topical Application: In Vitro Photoprotective, Antioxidant and Anti-Melanoma Activities
Source: Pharmaceuticals (Basel). 2024 Jun 2;17(6):721. doi: 10.3390/ph17060721 (PMC11206566; doi:10.3390/ph17060721)
Supplement: Supplementary file 1 [file pharmaceuticals-17-00721-s001.zip › pharmaceuticals-2986378-supplementary.pdf]

## Supplementary Material

# Nanoemulsified essential oil of the *Melaleuca leucadendron* leaves for topical application: *in vitro* photoprotective, antioxidant and anti-melanoma activities

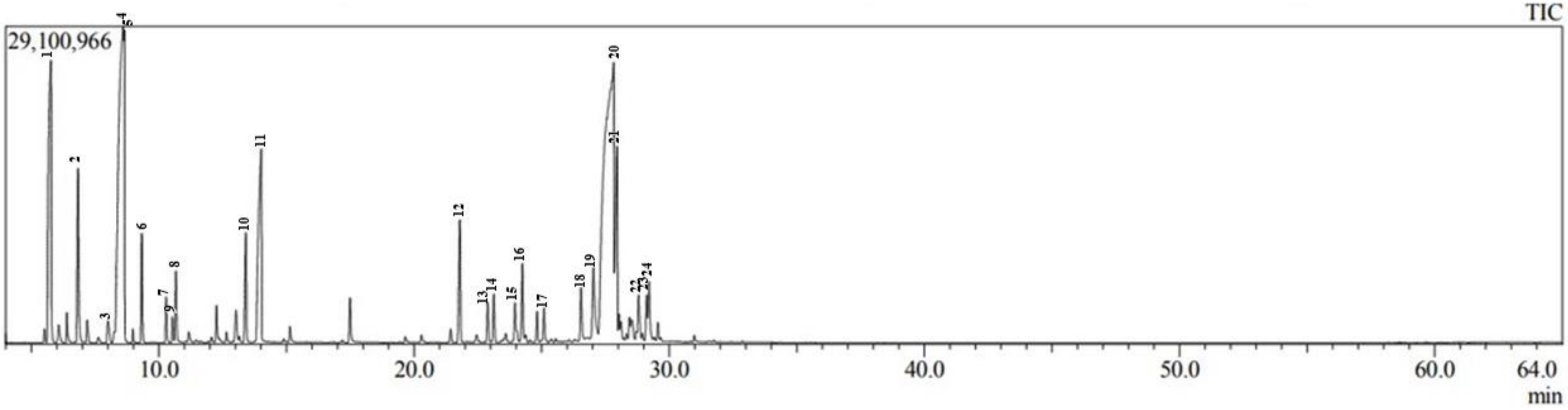


**Figure S1:** Chromatogram of the major chemical constituents of the essential oil extracted from *Melaleuca leucadendron* leaves: (1) α-pinene; (2) β-pinene; (3)(+)-2-Carene; (4) β-terpineol; (5) Eucalyptol; (6) γ-terpinene; (7) Cyclohexene, 4-methyl-3-(1-methylethylidene)-; (8) Linalool; (9) Isopulegol; (10) Terpinen-4-ol; (11) α-terpineol; (12) Caryophyllene; (13) α-caryophyllene; (14) Aromadendrene; (15) β-selinene; (16) Ledene; (17) δ-cadinene; (18) Palustrol; (19) Caryophyllene epoxide; (20) Hinesol; (21) Viridiflorol; (22) α-cadinol; (23) β-selinenol; (24) Selinenol.
